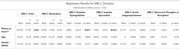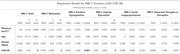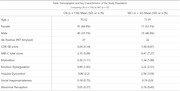# Relationship of Plasma *p*‐tau217 and Mild Behavioral Impairment in Elderly Individuals Without Dementia

**DOI:** 10.1002/alz70856_107716

**Published:** 2026-01-22

**Authors:** Ana Paula Bernardes Real, Arthur C. Macedo, Wyllians Vendramini Borelli, Tevy Chan, Nesrine Rahmouni, Seyyed Ali Hosseini, Etienne Aumont, Joseph Therriault, Gleb Bezgin, Wan Lu Jia, Brandon J Hall, Stuart William Mitchell, Jenna Stevenson, Lydia Trudel, Anna Marier, Elise Levinoff, José A Morais, Pedro Rosa‐Neto

**Affiliations:** ^1^ McGill University, Montreal, QC, Canada; ^2^ Montreal Neurological Institute, Montréal, QC, Canada; ^3^ Department of Geriatrics, McGill University, Montreal, QC, Canada; ^4^ Translational Neuroimaging Laboratory, The McGill University Research Centre for Studies in Aging, Montréal, QC, Canada; ^5^ McGill University Research Centre for Studies in Aging, Montreal, QC, Canada; ^6^ Montreal Neurological Institute, Montreal, QC, Canada; ^7^ Universidade Federal do Rio Grande do Sul, Porto Alegre, Rio Grande do Sul, Brazil; ^8^ Brain Institute of Rio Grande do Sul (InsCer), PUCRS, Porto Alegre, Rio Grande do Sul, Brazil; ^9^ The McGill University Research Centre for Studies in Aging, Montreal, QC, Canada; ^10^ Université du Québec à Montréal, Montréal, QC, Canada; ^11^ University of Montreal, Montreal, QC, Canada; ^12^ Douglas Research Centre, McGill University, Montreal, QC, Canada; ^13^ McGill University Health Centre, Montreal, QC, Canada; ^14^ Translational Neuroimaging Laboratory, Montreal, QC, Canada

## Abstract

**Background:**

Mild Behavioral Impairment (MBI) involves the late‐onset, sustained emergence of neuropsychiatric symptoms (NPS) in predementia populations. Prior studies examining amyloid and tau PET in relation to MBI have shown a link to amyloid burden but not to tau. However, the role of plasma biomarkers in MBI remains uncertain. In this study, we aimed to determine whether MBI is associated with plasma *p*‐tau217 levels in older adults without dementia.

**Method:**

We included 168 older adults (136 cognitively unimpaired [CU] and 32 with mild cognitive impairment [MCI] ) from the TRIAD cohort. Participants had amyloid status assessed by [18F]AZD4694 Aβ‐PET. Plasma *p*‐tau217 levels were quantified using the Janssen Simoa Assay. MBI was assessed using the Mild Behavioral Impairment Checklist (MBI‐C), global cognition was measured with the Mini‐Mental State Examination (MMSE) and the sum of boxes of the Clinical Dementia Rating (CDR‐SB). Multivariable linear regression analyses examined associations between plasma *p*‐tau217 and MBI‐C total and subdomain scores, adjusting for age, sex, and CDR‐SB.

**Result:**

When controlling for age and sex, higher *p*‐tau217 levels were significantly associated with increased MBI‐C total (β = 15.97, *p* =  0.03), emotion dysregulation (β = 5.98, *p* =  0.017), and impulse dyscontrol (β = 7.92, *p* =  0.012). However, these associations did not remain significant once CDR‐SB was included in the model. Instead, MBI‐C total and its subdomains emotion dysregulation, impulse dyscontrol, and motivation were related only to cognitive status.

**Conclusion:**

These findings suggest that *p*‐tau217 could play a role in MBI, particularly in the impulse dyscontrol and emotion dysregulation domains, neuropsychiatric symptoms which have been previously linked to Alzheimer's disease. However, overall cognitive status emerged as the strongest predictor of these behavioral symptoms in older adults. Further research is warranted to clarify how *p*‐tau217 levels and cognitive decline interact to influence the risk and progression of MBI.